# Limit of detection and limit of quantification development procedures for organochlorine pesticides analysis in water and sediment matrices

**DOI:** 10.1186/1752-153X-7-63

**Published:** 2013-04-05

**Authors:** Naghmeh Saadati, Md Pauzi Abdullah, Zuriati Zakaria, Seyedeh Belin Tavakoli Sany, Majid Rezayi, Houshang Hassonizadeh

**Affiliations:** 1 School of Chemical Sciences and Food Technology, Faculty of Science and Technology, Universiti Kebangsaan of Malaysia, Bangi, Malaysia; 2 Water, Soil and Sediment Laboratories Center, Khuzestan Water and Power Authority, Ahvaz, Iran; 3 Centre for Water Research and Analysis (ALIR), Universiti Kebangsaan of Malaysia, Bangi, Malaysia; 4 Malaysia-Japan International Institute of Technology, Universiti Technologi Malaysia, Jalan Semarak, Kuala Lumpur; 5 Institute of Biological Sciences University of Malaya, Kuala Lumpur, 50603, Malaysia; 6 Khuzestan Water and Power Authority, Ahvaz, Iran

**Keywords:** LOD, LOQ, OCPs, SPE, Soxhlet, Water, Sediment

## Abstract

**Background:**

Reliable values for method validity of organochlorine pesticides determination were investigated, in water by solid phase extraction and in sediment by Soxhlet extraction, followed by gas chromatography equipped with an electron capture detector. Organochlorine pesticides are categorized as Persistent Organic Pollutants. Hence, critical decisions to control exposure to these chemicals in the environment are based on their levels in different media; it is important to find valid qualitative and quantitative results for these components. In analytical chemistry, internal quality procedures are applied to produce valid logical results.

**Result:**

In this study, 18 organochlorine pesticides were targeted for analysis and determination in water and river sediment. Experiments based on signal-to-noise ratio, calibration curve slope and laboratory fortified blank methods were conducted to determine the limits of qualification and quantification. The data were compared with each other. The limitation values, following Laboratory Fortified Blank, showed significant differences in the signal-to-noise ratio and calibration curve slope methods, which are assumed in the results for the sample concentration factor to be 1,000 times in water and 10 times in sediment matrices. The method detection limit values were found to be between 0.001 and 0.005 μg/L (mean of 0.002 ± 0.001) and 0.001 and 0.005 μg/g (mean of 0.001 ± 0.001). The quantification limits were found to be between 0.002 and 0.016 μg/L (mean of 0.006 ± 0.004) and 0.003 and 0.017 μg/g (mean of 0.005 ± 0.003 μg/L) for water and sediment, respectively, based on the laboratory fortified blank method. Because of different slopes in the calibration methods, it was also found that the limitation values for some components from the internal standard were higher than from external standard calibration, because in the latter a factor for injection efficiency is applied for calibration.

**Conclusion:**

Technically, there are differentiations between detection limits for quality and quantity from component to component, resulting from noise, response factors of instruments and matrix interference. However, the calculation method is the cause of differentiation for each component of the different methods. The results show that for no matter what component, the relationship between these levels in different methods is approximately: Signal to Noise : Calibration Slope = 1:10. Therefore, due to different methods to determine LOD and LOQ, the values will be different. In the current study, laboratory fortified blank is the best method, with lower limitation values for Soxhlet and solid phase extraction of OCPs from sediment and water, respectively.

## Background

In practice, the method development step is the primary step in the analyses. The differences between the data collected by different chemists are caused by differences in analytical method development. Environmental conditions, differences in equipment and reference values are factors causing differences in data. These variations in data are acceptable at a restricted level, which is defined in the method development step. One of the most important steps in method development involves determination of the limits of qualification and quantification. Hence, there are various methods to define these parameters, namely an instrumental detection limit, a limit of detection, a method detection limit and a method quantification limit. These are some of those used in analytical chemistry.

The quality of data varies due to changes in reagents, equipment, testing utilities, calibration methods, operators and/or analysts. Because of this, Quality Control and Quality Assurance measures were established to ensure the integrity of results. In this way, analytical practices need to use results which are qualified by validation methods. Users are keen on some indication of the quality of results, which are shown by method validation. Limits of detection (LOD) and of quantification (LOQ) are the most important values that researchers look for when considering method validity. Among the different methods to measure LOD and LOQ, an analyst should select an appropriate one. Problems with the detection and quantification of an analyte can result from matrix effects, sample concentration or other conditions, such as instrument sensitivity and reagent purity.

With regard to organochlorine pesticide analyses, the current practice for limits of detection and quantification was determined as part of the method development and validation. The LOD and LOQ were derived via three different methods, including: signal-to-noise ratio (SN), calibration curve slope (CCS) and laboratory fortified blank (LFB). The CCS method was studied in two procedures for internal standard calibration (CCSI) and external standard calibration (CCSE). These abbreviations are used in the text.

The subject of detection limits in analytical chemistry has improved since the 1970s [1-5]. The lowest concentration level at which a measurement is quantitatively meaningful is called the limit of quantitation (LOQ). This is most often defined as 10 times the signal-to-noise ratio. If the noise approximates to the standard deviation of the blank, the LOQ is 10 times the standard deviation of the blank. Based on American Public Health Association [6], recovery criteria are between 50% and 150%, with%RSD values of < = 20%.

Analytical methods are expected to have a linear dynamic range (LDR) of at least two orders of magnitude, although shorter ranges are acceptable [7]. Moreover, considering all these, recovery in the sample preparation method is an important parameter that affects quantitative issues, such as LOD, sensitivity, LOQ and even LOL (limit of linearity). Sample preparation techniques can enhance the performance results for better recovery, increased sensitivity and lower detection limits. The merit figures for some analytical methods of previous studies are gathered together in Table 1 for sediment matrices and in Table 2 for water matrices.

**Table 1:** Analytical figures of merit for organochlorine pesticide analysis in previous studies of sediment matrices

**Detection limit(ng/g)**	**Quantification limit(ng/g)**	**Recovery**	**Sample size (g)**	**Reference**	**Extraction method**	**Linearity μg/L**	**Analyze method**
0.01–1.55	–	63–115	25	[22]	SE^1^	–	GC-ECD
0.01–0.08	–	83.7 ±3.1	15	[28]	SE^1^	–	GC-ECD
0.02–0.16 (ng/kg)	–	–	–	[29]	SE^1^	–	GC-ECD
0.02–0.04	–	90–110	20–25	[31]	SE^1^	–	GC-ECD
0.6–2.1	–	74–97.5	10	[30]	SE^1^	–	GC-ECD
0.01–0.05	–	94–97¤	15	[27]	SE^1^	–	GC-ECD
0.1–1	–	–	1	[37]	USE^2^	–	GC-ECD
0.1–0.2	0.2	72–121	20	[38]	USE^2^	–	GC-EI-MS
0.1	0.3	–	5	[39]	ASE^3^	0–250	GC-ECD

1) Soxhlet extraction, 2) Ultrasonic solvent extraction, 3) Accelerated solvent extraction.

**Table 2:** Analytical figures of merit for organochlorine pesticide analysis in previous studies of water matrices

**LOD (ng/L)**	**LOQ (ng/L)**	**Recovery**	**Sample size (L)**	**Reference**	**Extraction method**	**Linearity μg/L**	**Analyses method**
0.04–0.17	–	79.5 ± 8.2	2	[28]	SPE^1^	–	GC-ECD
5–35	15–106	70–130	1	[15]	SPE^1^	–	GC-ECD
0.6–3	–	78–95	1	[19]	SPE^1^	–	GC-ECD
0.08–0.16	–	75–87	1	[13]	SPE^1^	–	GC-ECD
0.0005–0.015	–	70–103	1	[14]	SPE^1^	–	GC-ECD
10–100	–	91–104.	1	[26]	LLE^2^	–	GC-ECD
0.01–1.03	–	85–105¤¤	-	[10]	LLE^2^	–	GC-ECD
5.5–20.6	–	71–101	1	[30]	LLE^2^	–	GC-ECD
1	–	79–96¤¤¤	1	[9]	LLE^2^	–	GC-ECD
1–3	5–12	81–95	1	[11]	LLE^2^		GC-MS

* linearity ng/g, ¤ recovery percentage based on Surrogate Standard, ¤¤ recovery for HCH and DDT, ¤¤¤ Internal standard recovery, 1) solid phase extraction, 2) liquid-liquid extraction.

Trace analysis, as a field of study encompassing pesticide residue analysis, has made considerable advances regarding selectivity and detection limits. From the 1940s to the early 1950s, the mainstream trace analysis techniques were gravimetric and bioassay trace analysis methods that extended the detection limits to almost 1 ppm, which was the maximum level at that time [8]. Today, there are several new extraction methods, such as: liquid-liquid extraction (LLE) [9-11], solid-phase extraction (SPE) [12-15], solid-phase micro-extraction (SPME) [16], stir-bar sorptive extraction (SBSE) [17], selective pressurized liquid extraction (SPLE) [18], Soxhlet extraction (SE) [9,19-30], ultrasonic extraction (USE) [31-38], microwave-assisted extraction (MAE) and accelerated solvent extraction (ASE) [39]; and detection limits have improved to very low levels. Sonication and Soxhlet extraction work well for chlorinated pesticides and PCBs. Sonication is a faster technique, but requires constant operator attention. In both techniques, problems such as contamination are attributed to either contaminated reagents, especially sodium sulfate, or poor laboratory practices being used when transferring sample extracts [40].

On the other hand, accelerated solvent extraction (ASE) offer fast extraction (12–18 min.) with a small amount of solvent (15–40 mL) and a large sample (up to 100 g). However, ASE equipment is relatively expensive and extraction normally requires a cleanup step. SPE has been used to extract pesticide and herbicide compounds from aqueous samples [41]. In general, the biggest drawbacks with SPE are plugging the disk or tube with suspended solids and the breakthrough of targeted organics; therefore, this extraction method works most reliably if contamination levels and solids are low [42]. SPE allows very fast extraction and low solvent volumes.

Soxhlet extraction is named after Baron Von Soxhlet, who invented this method in the mid-nineteenth century [43]. This procedure is widely used for extracting non-volatile and semi-volatile organic compounds from solids, such as sediment, soils, sludge and waste [44]. The Soxhlet extraction process ensures intimate contact of the sample matrix with the extraction solvent. It is applicable to the isolation and concentration of water-insoluble and slightly water-soluble organics, in readiness for a variety of chromatographic procedures. Since the sample is extracted with cooled, condensed solvents, Soxhlet is slow and can take between 6 to 48 hours. The extract volume is relatively large, so a solvent evaporation step is usually needed to concentrate the analyte prior to cleanup and analysis. The sample size is usually 10 g or more. Multiple samples can be extracted in separate Soxhlet units, and the extraction can run unattended. Soxhlet is a powerful well-established technique. Compared with other methods, Soxhlet is something of a benchmark technique as few parameters can affect the extraction. The main disadvantages of this method are the long extraction time and the relatively large solvent consumption [43,45].

## Result and discussion

**Figure 1:**
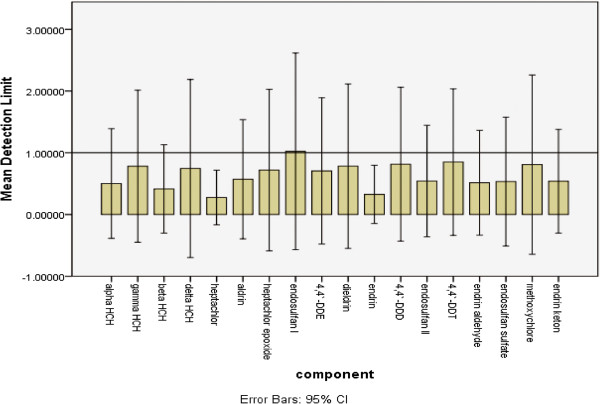
Detection Limit (ng/L) error bar for water matrix.

All the results for detection limits for water matrices were gathered together and are shown in Figure 1, i.e. an error bar graph for the mean of detection limits of each component shown separately.

### Detection limits based on signal to noise ratios (SN)

The signal and noise heights are used to calculate signal-to-noise ratio. Clearly, lower values for noise height and higher values for signal height result in lower values for detection limits. This approach is mostly recommended when the instrument exhibits noise in the absence of an analyte. In the guidelines for the validation procedure of Harmonization [46], signal-to-noise ratio it is suggested to apply analytical procedures that exhibit baseline noise. In instrumental analysis this is considered the detection limit, because in instrumental analysis, such as chromatography, the response of the instrument is strongly related to all the instrumental parts' properties taken together, e.g. injection port, column, oven, detector, user, etc. Signal-to-noise ratio (S/N) is calculated by 2H/h, where: H is the height of the peak corresponding to the component concerned, in the chromatogram obtained from the prescribed reference solution, measured from the maximum of the peak to the extrapolated baseline of the signal observed from a distance equal to 20 times the width at half-height; h is the range of the background noise in a chromatogram obtained after the injection or application of a blank, observed from a distance equal to 20 times the width at half-height of the peak in the chromatogram obtained with the prescribed reference solution and, if possible, situated equally around the place where this peak is found. According to this formula, detecting a signal produced by an analyte in its standard form leads to the instrumental detection limit, without considering any steps in the sample treatment. Therefore, in a multi-residue determination such as the current study, each analyte shows its response free of any interfering factor, since it is just a standard solution, and this is what is generally known as the instrumental detection limit (IDL).

Table 3 illustrates the maximum noise value for 4,4`-DDT (520) as the highest value of detection limit (0.563 μg/L in water). Detection limits ranged from 0.066 to 0.563 μg/L in water (mean of 0.202±0.141μg/L); meanwhile, noise and signal height ranged from 86 to 520 (mean of 205±126) and from 1526 to 5116 (mean of 3411±997), respectively, for 2 μg/L of fortified matrix water blank. An increasing trend in detection limit values appeared in the order: Heptachlor epoxide< β-HCH< δ-HCH< α-HCH< heptachlor< aldrin< endosulfan II< 4,4'–DDE< dieldrin< endrin< methoxychlor< endrin aldehyde< γ–HCH < 4,4'-DDD< endosulfan I< endosulfan sulfate< endrin ketone< 4,4'-DDT. Indeed, SN indicates the instrument performance in the desired analytes. So the most important characteristics in chromatography performance are selected detector (ECD), stationary phase (column, HP-5ms), mobile phase (carrier gas, N2), injection mode (splitless) and the temperatures of injection port, oven and detector.

**Table 3:** Estimated method detection limit values

			**LOD (μg/L)**				**LOQ (μg/L)**			
**Component**	**NH**^**a**^	**SH**^**b**^	**S/N ≈ 3**	**LFB**^**c**^	**IS CAL**^**d**^	**EX CAL**^**e**^	**S/N ≈ 10**	**LFB**^**c**^	**IS CAL**^**d**^	**EX CAL**^**e**^
α-HCH	149	5116	0.087	0.00211	1.18	0.74	0.29	0.0067	3.94	2.47
γ -HCH	148	1895	0.234	0.00173	1.32	1.66	0.78	0.0055	4.39	5.54
β-HCH	103	4670	0.066	0.00217	0.68	0.89	0.22	0.0069	2.28	2.95
δ-HCH	104	4367	0.071	0.00462	1.81	0.90	0.24	0.0147	6.04	3.00
Heptachlor	128	4284	0.090	0.00487	0.71	0.30	0.30	0.0155	2.38	0.98
Aldrin	125	3872	0.097	0.00245	1.04	1.26	0.32	0.0078	3.47	4.20
Endosulfan I	86	3938	0.066	0.00321	1.86	1.08	0.22	0.0102	6.19	3.61
Heptachlor epoxide	476	4336	0.329	0.00233	2.04	2.11	1.10	0.0074	6.81	7.02
4,4'-DDE	163	3636	0.134	0.00097	1.44	0.96	0.45	0.0031	4.81	3.19
Dieldrin	161	3205	0.151	0.00139	1.34	1.67	0.50	0.0044	4.48	5.57
Endrin	160	2789	0.172	0.00115	0.66	0.73	0.57	0.0037	2.19	2.45
4,4'-DDD	337	3597	0.281	0.00181	1.38	1.46	0.94	0.0058	4.60	4.85
Endosulfan II	97	2319	0.125	0.00112	1.25	1.33	0.42	0.0036	4.18	4.42
4,4'-DDT	520	2773	0.563	0.00168	1.47	1.42	1.88	0.0054	4.89	4.72
Endrin aldehyde	181	2909	0.187	0.00097	0.49	1.11	0.62	0.0031	1.64	3.69
Endosulfan sulfate	301	2394	0.377	0.00066	0.18	1.10	1.26	0.0021	0.59	3.68
Methoxychlor	229	3780	0.182	0.00131	1.01	2.08	0.61	0.0042	3.36	6.93
Endrin ketone	217	1526	0.427	0.00102	1.35	1.16	1.42	0.0033	4.49	3.85
Max	520	5116	0.563	0.005	2.043	2.107	1.875	0.016	6.809	7.024
Min	86	1526	0.066	0.001	0.178	0.295	0.218	0.002	0.593	0.984
Average	205	3411	0.202	0.002	1.179	1.219	0.674	0.006	3.929	4.062
RSD	126	997	0.141	0.001	0.493	0.466	0.472	0.004	1.645	1.552
%CV	62	29	70	61	42	38	70	61	42	38

^a^ Noise Height(2 μg/L),^b^ Signal Height(2 μg/L), ^c^ LFB:Laboratory Fortified Blank(10 μg/L),^d^ and ^c^ internal and external calibration respectively.

### Detection limits based on the slope of calibration curve (CCS)

The slope of a calibration curve is another procedure to assume a limit of detection in analytical chemistry. Rajaković and Marković [47] classified the calibration curve detection limits into three groups: ordinary least square, weighted least square and nonlinear calibration curves. The results in Table 4 are based on linear least square. Two calibration methods are used for quantification, namely internal and external calibration. The detection limits were calculated based on the slopes of these two plots. The responses of analytes (y) were plotted against the concentrations of a series of standard values of the analytes (x). The detection limit was calculated by the equation: $a+\frac{3\times SD_{y}}{\text{slope}}$, (where a is a calibration equation intercept) [47,48]. For external calibration (CCSE), detection limits ranged from 0.295 to 2.107 μg/L in water (mean of 1.219±0.466 μg/L). Fatoki and Awofolu [30] determined values between 5.5 and 20.6 ng/L, based on a linear calibration curve equation for some organochlorine pesticides in water samples. Meanwhile, in a study on an arsenic detection limit using ICP, Rajaković and Marković [47] shows unreliable detection limit values based on a linear least square calibration curve equation.

**Table 4:** External and internal calibration data for 18 target organochlorine pesticides

**Component**	**Linear range (μg/L)**	External calibration*			Internal calibration**		
		**Equation**	**Slope**	**R**^**2**^	**Equation**	**Slope**	**R**^**2**^
α–HCH	1.95-62.5	Y = 13.7X-2.04	13.74	0.9996	Y = 1.8X + 0.71	1.84	0.9994
γ–HCH	1.95-62.5	Y = 4.6X + 6.01	4.6327	0.9990	Y = 0.6X + 1.20	0.62	0.9987
β–HCH	1.95-62.5	Y = 12.9X-2.72	12.90	0.9996	Y = 1.7X + 0.53	1.73	0.9998
δ–HCH	1.95-62.5	Y = 13.1X-7.36	13.15	0.9989	Y = 1.8X-0.09	1.76	0.9982
4,4'-DDE	1.95-62.5	Y = 10.1X + 10.21	10.03	0.9988	Y = 1.3X + 2.21	1.34	0.9991
4,4'-DDD	1.95-62.5	Y = 8.6X + 10.91	8.67	0.9988	Y = 1.2X + 2.22	1.16	0.9988
4,4'-DDT	1.95-62.5	Y = 7.2X + 9.99	7.21	0.9987	Y = 1.0X + 1.98	0.97	0.9984
Heptachlor	1.95-62.5	Y = 12.4X + 6.26	12.36	0.9999	Y = 1.6X + 1.83	1.65	0.9995
Heptachlor epoxide	1.95-62.5	Y = 10.6X + 14.67	10.66	0.9993	Y = 1.4X + 2.92	1.42	0.9988
Endosulfan I	1.95-62.5	Y = 10.0X + 16.38	10.05	0.9980	Y = 1.3X + 3.11	1.34	0.9980
Endosulfan II	1.95-62.5	Y = 6.3X + 8.96	6.34	0.9989	Y = 0.8X + 1.75	0.85	0.9995
Endosulfan sulfate	1.95-62.5	Y = 7.5X- 4.44	7.45	0.9990	Y = 1.0X-0.14	1.00	0.9999
Aldrin	1.95-62.5	Y = 11.2X + 9.37	11.22	0.9995	Y = 1.5X + 2.18	1.50	0.9993
Dieldrin	1.95-62.5	Y = 9.2X + 11.67	9.19	0.9986	Y = 1.2X + 2.37	1.22	0.9989
Endrin	1.95-62.5	Y = 8.3X + 3.35	8.30	0.9997	Y = 1.1X + 1.10	1.11	0.9996
Endrin aldehyde	1.95-62.5	Y = 7.6X + 2.88	7.61	0.9992	Y = 1.0X + 0.97	1.02	0.9998
Methoxychlor	1.95-62.5	Y = 8.8X + 11.11	8.87	0.9984	Y = 1.2X + 2.26	1.18	0.9988
Endrin ketone	7.81-62.5	Y = 3.1X + 3.51	3.13	0.9992	Y = 0.4X + 0.78	0.42	0.9998

*Y = Standard Area, C = standard Concentration ** Y = standard Area × internal standard concentration/internal standard area, C = Standard Concentration.

On the other hand, by internal standard calibration, detection limits (CCSI) were found to range from 0.178 to 2.043 (mean of 1.179±0.494). An increasing trend in detection limit values appeared in the order: endosulfan sulfate> endrin aldehyde> 4,4'-DDE> endrin ketone> endosulfan II> endrin> methoxychlor> dieldrin> 4,4'-DDT> γ-HCH> endosulfan I> 4,4'-DDD> α-HCH> β-HCH> aldrin> heptachlor epoxide> δ-HCH> heptachlor. Thus, the lowest detection limit was for endosulfan sulfate, and the highest for heptachlor, based on CCS. In the case of CCSE, the increasing trend for the limit of detection is: heptachlor> endrin> α-HCH> β-HCH> δ-HCH> 4,4'-DDE> heptachlor epoxide> endosulfan sulfate> endrin aldehyde> endrin ketone> aldrin> endosulfan II> 4,4'-DDT> 4,4'-DDD> γ-HCH> dieldrin > methoxychlor> endosulfan I. Therefore, the increasing trend for the two methods (CCSI and CCSE) is not the same, which could be the result of a relative response factor coming from an internal standard effect. In addition, detection limits derived from an internal standard calibration slope show lower levels for detection limits derived by CCSE. However, data analyses by SPSS showed no significant differences between the detection limits of these two methods.

### Detection limits based on laboratory fortified blank (LFB)

The laboratory fortified blank-based detection limit presents another range of detection limits for target organochlorine pesticides, based on standard deviation and T-value [49,50]. The detection limits based on LFB ranged from 0.001 to 0.005 μg/L in water (mean of 0.002±0.001 μg/L). This range of detection limits is in agreement with Darko and Akoto [19], who determined organochlorine pesticide residues in water from Lake Bosomtwi, Ghana. However, this range does not support the studies of Tan and He [28] and Samoh and Ibrahim [15], who reported the lower and higher ranges for detection limits, respectively.

The detection limit increasing trend is in the order: endosulfan sulfate> 4,4'-DDE> endrin aldehyde> endrin ketone> endosulfan II> endrin> methoxychlor> dieldrin> 4,4'-DDT> γ -HCH> endosulfan I> 4,4'-DDD> α-HCH> β-HCH> aldrin> heptachlor epoxide > δ-HCH> heptachlor. The lowest and highest values for detection limits were for endosulfan sulfate and heptachlor, respectively. A laboratory fortified blank takes into account all steps of sample preparation and analysis.

Detection limits based on LFB for sediment are shown in Table 5. The values show detection levels between 0.001 and 0.005 ng/g (mean of 0.001±0.001). This range is similar to the results of Kim and Kang [51] ( 0.002 to 0.005 ng/g). The results of Tan and He [28] (0.01–0.08 ng/g) and Kim and Lee [29] (0.02–0.16 ng/kg) indicate higher and lower levels, when comparing with this study's detection limits, respectively

**Table 5:** LOD and LOQ in sediment* (ng/g)

**Component**	**Average**	**SD**	**LOD = SD × T **^**a**^	LOQ = 10*SD
α-HCH	0.0048	0.0003	0.001	0.003
γ –HCH	0.0053	0.0005	0.002	0.005
β-HCH	0.0052	0.0017	0.005	0.017
δ-HCH	0.005	0.0006	0.002	0.006
4,4'-DDE	0.0059	0.0006	0.002	0.006
4,4'-DDD	0.0046	0.0007	0.002	0.007
4,4'-DDT	0.0049	0.0003	0.001	0.003
Heptachlor	0.0066	0.0003	0.001	0.003
Heptachlor epoxide	0.0041	0.0004	0.001	0.004
Endosulfan I	0.0063	0.0003	0.001	0.003
Endosulfan II	0.0054	0.0003	0.001	0.003
Endosulfan sulfate	0.0044	0.0004	0.001	0.004
Aldrin	0.0054	0.0003	0.001	0.003
Dieldrin	0.0063	0.0003	0.001	0.003
Endrin	0.0045	0.0003	0.001	0.003
Endrin aldehyde	0.0051	0.0003	0.001	0.003
Methoxychlor	0.0046	0.0004	0.001	0.004
Endrin ketone	0.0055	0.0004	0.001	0.004
Max			0.005	0.017
Min			0.001	0.003
Average			0.001	0.005
SD			0.001	0.003
%CV			68	71

*Based on LFB: 1 mL mix of standard with 0.060 ppm into 10 g of sediment is equal to 0.006 mg/g, ^a^T-value = 3.14 for 7 replicates.

### Comparisons between the three methods of detection limit calculation

Method detection limits in the current study consist of three methods that are documented in analytical chemistry, and which to use is a chemist’s decision. In the signal-to-noise ratio method, the emphasis is on instrumental properties. In the CCS method, attention is paid to a fast and initial assumption for the detection limit. The LFB method makes an assumption based on all methods' procedures, whether they improve or reduce detection values. The aim of finding the differences between these data is to reveal patterns in the data, to see whether they are reliable for reporting detection limit values. Miller and Miller [48] recommends comparing different methods to find reliable detection limit values. On the other hand, depending on the nature of each method, care should be taken by the chemist when using any of them. It is not recommended to use the calibration curve method for single point calibrations [48]. The signal-to-noise procedure is mostly used for IDL, rather than for a method detection limit. LFB can produce high values in cases of increasing interference in several steps of a sample treatment technique. Among the three calculation methods, detection limits based on LFB showed lower values.

Considering both internal standard and external standard as one calculation method, the increasing trend in detection limits is as follows: LFB<SN<CCS. Although signal to noise should have the lowest instrumental detection limit and the LFB method should be higher than that, because it has worse sensitivity due to interference, this finding shows that an analyst can improve the method detection limits in case of a limitation in IDL. Chung and Chen [52] also mentioned this effect of achieving a method detection limit in a matrix caused by reducing the chemical noise from matrix extractions. Therefore, extraction by SPE and enhancement can achieve results with a lower detection limit when analyzing organochlorine pesticides, as shown in this study. Similarly, Janska and Lehotay [53] found lower detection limits in matrix extracts in his study on vegetables, as well.

The similarities among all the methods conducted and a casual inspection of them raises the possibility of having no significant differences between the data from the four methods (CCSI, CCSE, SN, LFB) statistically. Therefore, an independent sample t-test is appropriate for studying the differences associated with the methods applied to the 18 target organochlorine pesticides. An independent-sample t-test was conducted with SPSS to compare the detection limits (t(34)=9.5, P=0, two-tailed). There was a significant difference between SN-based detection limits (M=0.202, SD=0.14) and CCS values for CCSI and CCSE based on target OCPs. There were no significant differences between CCSI-based detection limits (M=1.12, SD=0.5) and CCSE-based detection limits (M=1.22, SD=0.45; t(34)=0.6, P=0.56, two-tailed). LFB- (M=0.005, SD=0.002) and SN-based (M=0.202, SD=0.14; t(34)=6, P=0.00, two-tailed) detection limits showed significant differences. An independent-sample t-test was also conducted to compare detection values based on CCSI and LFB. There was a significant difference between LFB-based detection limits (M=0.005, SD=0.002) and CCSI-based detection limits (M=1.12, SD=0.5; t(34)=-7.5, P=0.00, two-tailed). Similar results were found in the research of Rajaković and Marković [47] into different methods of detection limit calculation for arsenic by ICP. He mentions that a unique value for LOD calculated by a certain model cannot be directly compared to those calculated by other models.

## Conclusion

The smallest amount of analyte use in the case of particular samples consists of a very small amount of OCPs. The aim is to find a procedure that offers the capability of detecting the lowest amount of analyte. This subject is one of the concerns of OCP analytical methods for environmental samples. A study was conducted to investigate the reliability of LOD using the SPE and Soxhlet methods to analyze OCPs in water and sediment samples. In this study, it was shown that LOD and LOQ are different in terms of quantity using different methods. Considering the results of the data analysis and the pattern of samples applied to assume detection limits, LFB (i.e. laboratory fortified blank) is the one with the lowest values of detection limit and is also reliable. α-HCH determination by GC-ECD indicates significant differences between signal-to-noise ratio and laboratory fortified blank with calibration curve slope methods. These differences are consequences and an effect of the analyzing procedures used. Therefore, an analyst could find the cause of the limitations on detection and quantification in order to admit, ignore, improve, modify or exchange a specific situation. Therefore, the lowest amounts of 0.006±0.004 μg/L and 0.005±0.003 μg/g (highest values of limit of quantification) of the target OCPs can be detected and quantified by the methods studied in water and sediment samples, respectively.

## Methods

### Reagent

The ampoule of mixed organochlorine pesticide standards consisted of α-HCH, β-HCH, γ-HCH, δ-HCH, 4,4´DDT, 4,4´DDE and 4,4´DDD and was obtained from Supelco (Belle-Fonte, USA). The stock solution (200 ppm) of mixed OCPs was prepared in 10 mL of n-hexane. Fresh working standard solutions containing a mixture of the mixed OCP, surrogates (2, 4, 5, 6-tetrachloro-m-xylene & decachlorobiphenyl) and the internal standard component (pentachloronitrobenzene) were prepared by stepwise dilution of the stock solution with the range 1.95, 3.91, 7.81, 15.63, 31.25 and 62.5 μg/L. Organic free water was prepared by passing distilled water through a filter bed containing about 250 g of activated carbon [6,54] and stored in a cleaned narrow-mouth bottle with Teflon septa and a screw cap. All glassware was rinsed with analytical n-hexane before use. All the solvents which were used for extraction, cleanup and enhancement were pesticide grade. The anhydrous sodium sulphate was purified by heating it to 400°C for 4hrs. Florisil (PR Grade) was used for cleanup in an activated form [55]. Disposable 6 mL SPE cartridges with 0.5 gr sorbent-octadecyl bonded, endcapped silica UCT, ENVIROCLEAN EEC08156 were used to extract water samples

### Sample collection

The sediment samples were collected with a Peterson grab sampler to a depth of about 5 cm. The sediment samples were wrapped in aluminum foil and stored at 4°C until analysis. 250 g of the sediment was collected from each station to determine particle size. Water samples were collected in glass bottles. The samples were kept at 4°C prior to the extraction process. A multi-parameter portable device, (YSI 556 MPS-USA), was used for onsite measurements of the temperature, electrical conductivity, total dissolved solids, salinity and turbidity of the rivers.

### Quality control

**Figure 2:**
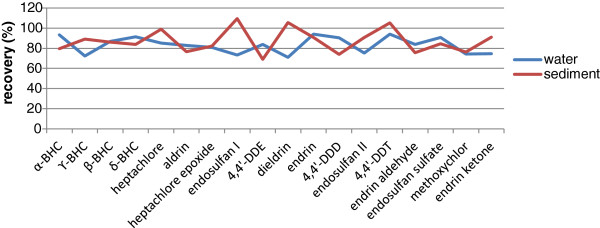
Average recovery values for the target OCPs in the water and sediment standard samples.

Gas chromatograph mass spectrometer (GC/MS) analyses were performed with an Agilent 7890A gas chromatograph (GC) directly coupled to the mass spectrometer system (MS) of an Agilent 5975C inert MSD with a triple-axis detector to confirm the order of components. The internal standard concentration was kept constant in all solutions at 100 μg/L. Relative response factors were applied to quantify data. Percentage recoveries were verified by the surrogate component. Surrogate standards were added to each sample to monitor extraction performance and matrix effects. A recovery value of between 75% and 125% was considered for quantification and 65% to 135% for qualification, as well. Figure 2 presents the average recovery values for the target OCPs in the water and sediment standard samples. The concentrations of the OCPs were not modified by the recovery ratios of the surrogates. Every sample was analyzed in triplicate, and the average amount was applied in the data analysis.

### Experimental procedures

#### Sediment

The sediment water content was determined by oven drying about 10 g of wet sediment for 12 h at 105°C. A series of mesh sieves ranging from 0.0125 mm to 64 mm were applied to determine the particle size of the sediment samples. 10.00 g of an air-dried grounded homogenized sediment sample was mixed with 10.00 g of anhydrous sodium sulfate, which was spiked with 1 mL of 0.160 ppm surrogate solutions extracted with 300 mL of n-hexane/acetone 50:50 for six hours in a Soxhlet extractor. The extracted volume was reduced using a Rotovap evaporator to about 5 mL. Then, the solution was loaded onto the Florisil column cleanup packed with 20.0 g of activated Florisil. The Florisil column was eluted three times with 65 mL of n-hexane, 45 mL of 70:30 n-hexane/dichloromethane and 55 mL of dichloromethane. The cleaned solution was concentrated by evaporating the solvent using a Rotovapor-R-3000 evaporator. This solution was further concentrated to 2 mL with a stream of high purity nitrogen. 1 μL of the concentrated solution was spiked with exactly 1 μL at 100 ppm of internal standard before injection into the GC-ECD.

#### Water

A 1,000 mL water sample was spiked with 1 mL of 0.080 ppm surrogate solution with 5 mL of methanol added and passed through a 6 mL capacity C18 cartridge. The cartridge was optimized with 5 mL of ethyleacetate, 5 mL of dichloromethane, 10 mL of methanol and 10 mL of organic free water before use. Then it was eluted with 5 mL of ethyleacetate and 5 mL of dichloromethane. This eluted solution was concentrated with a stream of nitrogen to 1 mL. 1 μL of the concentrated solution was spiked with exactly 1 μL at 100 ppm of internal standard before injection to the GC-ECD.

#### Apparatus

**Figure 3:**
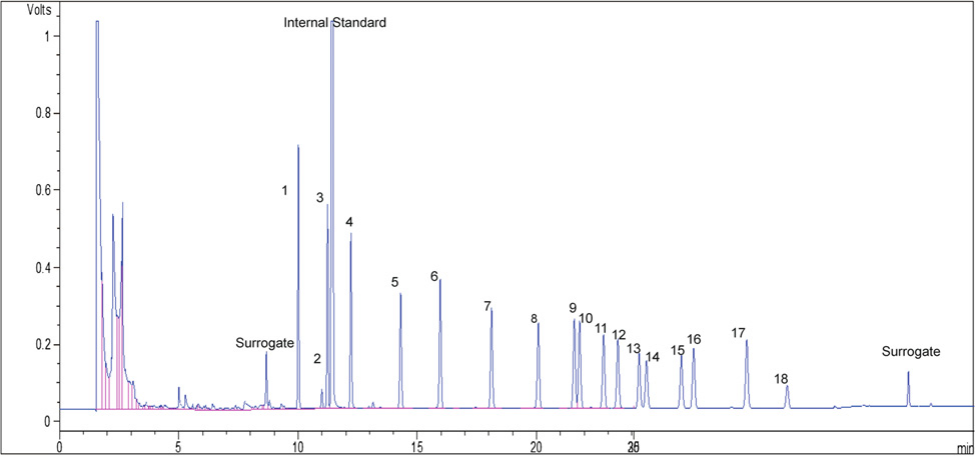
GC-ECD chromatogram of 18 OCPs, surrogates and internal standard.

A Varian chromopack CP-3800 Gas Chromatograph was applied to analyze the OCP in the samples. The instrument was equipped with a ^63^Ni electron capture detector and a 30 m × 0.32 mm i.d. (0.25 μm film thickness) HP-5 ms fused silica capillary column. Nitrogen gas was used as the carrier gas at 1.5 mL/min. The oven temperature was kept at 90°C for 1 minute and increased to 170°C at a rate of 3.5°C/min and then to 280°C at a rate of 5°C/min. The injector and detector temperatures were adjusted to 250°C and 300°C, respectively. 1 μL of each sample was injected into the GC-ECD for separation and quantitative analysis. Figure 3 shows a GC-ECD chromatogram of 18 OCPs, surrogates and internal standard. Peak identification was done based on GC/MS analyses. All 21 components, including 18 target OCPs and the internal standard and surrogates, were completely separated [56] by more than 6s, except peaks numbered 2 and 9 (Rs≈1). The symmetry factor of the peaks was between 0.9 and 1.3 in the European Pharmacopoeia range for chromatographic separation techniques. In Figure 3, the first component is after the first surrogate component and the last one is before the second. In this way, the analyst could be sure that all components were coming out of the column quantitatively between a bracket of two surrogate components, peak numbers 1 and 21 in Figure 3.

## Competing interests

The authors declare that they have no competing interests.
